# Single Printing Step Prussian Blue Bulk-Modified Transducers for Oxidase-Based Biosensors

**DOI:** 10.3390/bios13020250

**Published:** 2023-02-09

**Authors:** Darya Vokhmyanina, Elena Daboss, Olesya Sharapova, Mariia Mogilnikova, Arkady Karyakin

**Affiliations:** Chemistry Faculty of M.V. Lomonosov, Moscow State University, 119991 Moscow, Russia

**Keywords:** screen-printed electrodes, Prussian blue nanoparticles, biosensors, hydrogen peroxide, glucose, lactate

## Abstract

We report on hydrogen peroxide sensors made through a single printing step with carbon ink containing catalytically synthesized Prussian blue nanoparticles. Despite their reduced sensitivity, the resulting bulk-modified sensors displayed both a wider linear calibration range (5 × 10^−7^–1 × 10^−3^ M) and an approximately four times lower detection limit versus the surface-modified sensors due to the dramatically decreased noise resulting in, on average, a six times higher signal-to-noise ratio. The corresponding glucose and lactate biosensors demonstrated similar and even higher sensitivities compared to those of biosensors based on surface-modified transducers. The biosensors have been validated through analysis of human serum. The decreased time and cost for production of single printing step bulk-modified transducers, as well as their analytical performance characteristics, which are advantageous over conventional surface-modified ones, would be expected to enable their wide use in (bio)sensorics.

## 1. Introduction

Hydrogen peroxide remains a crucial analyte due to its widespread use as a bleaching, antiseptic, and disinfecting agent in such different fields as food processing, pharmaceutical research, clinical chemistry, textiles, the paper industry, etc. [1,2,3,4]. Additionally, H_2_O_2_ is the by-product of enzymatic processes involving oxidases. Reliable, accurate, specific, and sensitive methods for H_2_O_2_ determination continue to be widely investigated [5,6,7]. In addition to the above advantages, electrochemical sensors with relative simplicity, cost-effectivity, and time-saving and portability characteristics are a promising approach [8].

Electrochemical measurement using screen-printed electrodes (SPEs) is a well-known, simple, cost-effective, and convenient method to detect various molecules that also includes an excellent resource opportunity for miniaturization and mass production. Electrochemical sensing of hydrogen peroxide can be carried out by its oxidation or reduction processes, in which the latter has the advantage of low-potential measurement. This is especially important for the analysis of biological objects, as many presented reducing agents can affect accuracy when using high potentials. A lot of electrocatalysts are known for H_2_O_2_ reduction, among which Prussian blue (PB) has been proven to be the most advantageous one [9]. 

A variety of biosensors based on PB-modified SPEs and oxidase enzymes have been developed for uric acid [10], NADH [11], hypoxanthine [12], lactate [13], and glucose [14,15] detection. Such biosensors have detection limits in the micromolar range (0.4–10 µM), a wide linear range, and low operating potentials.

Even more effective for sensorics is the application of Prussian blue nanoparticles (PBNPs), also known as nanozymes (nanoparticles with enzyme-like activity). Developed in 2018 in our laboratory [16,17], PBNPs have been shown to possess even higher activity than peroxidase enzymes themselves. The mechanism of hydrogen peroxide reduction and substrate specificity was also investigated [18,19].

Glucose-6-phosphate [20], lactate [21], and glucose [22] biosensors based on PBNPs have been reported. For lactate biosensors, an almost twofold increase in sensitivity was observed compared to biosensors based on PB films [21]. The use of PBNPs in mixture with ITO made it possible to create not only an electrochemical, but also an optical sensor, which showed even lower detection limits [22]. The piezoelectric inkjet printing of PBNPs [23,24] allows for the advantageous analytical performance of H_2_O_2_ sensors: the detection limit is down to 0.2 µM [23], a linear range prolongs from 0 to 4.5 mM, and sensitivity is 762 mA·M^−1^·cm^−2^. A cholesterol biosensor based on these technologies and an immobilized cholesterol oxidase enzyme showed a detection limit of 0.2 mM [25]. However, achieving these characteristics requires printing up to 20 layers of PBNPs on each sensor, which seems quite complex and time consuming.

Though (bio)sensors based on SPEs modified with PBNPs proved to be very effective in terms of sensitivity, linear range, and selectivity, they still need the additional surface modification stage. We propose excluding this stage through the addition of PB nanozymes to carbon paste before screen printing working electrodes. Thus, hydrogen peroxide sensors can be produced by simple screen printing, which should be performed anyway, without any additional modifications. This idea will allow for easily scalable mass production of H_2_O_2_ sensors.

The use of PB-containing ink (Gwent C2070424P2) and even screen-printed sensors (DRP-710, Dropsens) [26,27,28] has been previously explored. Yet, the analytical performance of such (bio)sensors is usually not as good as that of sensors based on SPEs with a PB-modified surface. The linear range for a hydrogen peroxide sensor [26] is 0.03–5 mM with sensitivity of about 43 mA·M^−1^·cm^−2^, which is significantly lower than the characteristics of surface-modified sensors [21,23]. For uric acid detection, analytical characteristics are better for bulk-modified sensors than for surface-modified ones (linear range of 10–200 µM and a limit of detection of 3 µM [26] compared to 30–300 µM and 10 µM [10], respectively), probably due to different enzyme immobilization techniques. For lactate detection, a very narrow linear range (0.2–0.6 mM) was shown for lactates (dynamic range up to 4.4 mM) with sensitivity of about 36 mA·M^−1^·cm^−2^ [27], which is lower than the 200 mA·M^−1^·cm^−2^ (linear range 1–1000 µM) shown for biosensors based on PBNP surface-modified SPEs [21]. Previously, (bio)sensors with PB microparticles (average diameter 38 µm) bulk-modified carbon screen-printed electrodes with advanced characteristics were explored [29]: sensitivity of 137 and 3.21 mA·M^−1^·cm^−2^ and a limit of detection of 0.4 µM and 0.22 mM for a hydrogen peroxide sensor and glucose biosensor, respectively. Thus, using smaller Prussian blue particles results in improved sensor quality. 

Using bulk-modified electrodes leads to crucial decreases in time and labor costs through the simplification of mass production. However, commercial bulk-modified electrodes and inks are inferior in terms of analytical characteristics compared to surface-modified ones. We propose the use of Prussian blue nanoparticles as carbon/graphite ink modifiers, which has not been previously reported. This idea will allow for the development of a single-stage technology for the manufacture of hydrogen peroxide sensors with improved characteristics.

## 2. Materials and Methods

### 2.1. Reagents and Objects of Analysis

Experiments were carried out with Milli-Q water (18.2 MΩ·cm). Inorganic salts, hydrogen peroxide (30% solution), glucose, potassium lactate (60% solution), fructose, mannose, sodium gluconate, sodium tartrate, (3-aminopropyl)triethoxysilane (99%), and organic solvents were obtained from Sigma-Aldrich (Burlington, MA, USA) or Reachim (Moscow, Russia) at the highest purity. Perfluorosulfonated ionomer (PFSI) (10% solution in isopropyl alcohol), a structural analogue of Nafion, was obtained from Plastpolimer (St. Peterburg, Russia). Hydrochloric acid solutions were prepared from fixanals manufactured by Germed (Dresden, Germany).

Lactate oxidase (LOx, EC1.1.3.2) from Pediococcus species (Sorachim, Lausanne, Switzerland) was used in the form of a lyophilized protein with a declared activity of 32.8 U/mg. Glucose oxidase (GOx, EC 1.1.3.4) from Aspergillus niger (Sigma-Aldrich, Burlington, MA, USA) was used in the form of a lyophilized protein with a declared activity of 246.6 U/mg. Standard samples of blood serum were obtained from Spinreact (Girona, Spain).

### 2.2. Screen-Printed Electrode Fabrication

Screen-printed electrodes were produced with a SCF–300 (Technical Industrial Co. Ltd., Tsuen Wan, N.T. Hong Kong, China) screen printing device using carbon/graphite ink (C2030519P4, Sun Chemical, South Normanton, UK) for the working electrode and Ag conductive ink (PSP-2, NPP Delta-Pasty, Moscow, Russia) for the pseudo-reference and counter electrodes and contacts. The insulating layer was Uniplus Blue UPLX 51 from Unico (Ternat, Belgium); a DRT-230 UV-lamp (Lisma, Saransk, Russia) was used for curing. The substrate was a flexible polyethylene terephthalate film (250 µm thin) obtained from Vladimirskii Khimicheskii Zavod (Vladimir, Russia). The apparent geometric area of the working electrode was 0.0254 cm^2^.

For hydrogen peroxide sensor screen printing, Prussian blue nanoparticle (PBNP) suspensions with different PB concentrations (3.3–50 mM) were added to the carbon/graphite ink before its applying for working electrode printing. The PBNP concentration in the ink was 0.14–2.15 mg/g. Energy-dispersive X-ray spectroscopy was performed with EDAX, SDD Apollo XV (EDAX, Mahwah, NJ, USA).

Modification of the working electrode surface with PB to obtain control surface-modified blank electrodes was carried out on blank screen-printed electrodes that did not contain printed PBNPs (Figure S1). The current-free deposition of Prussian blue film was carried out from an equimolar mixture of iron (III) chloride and potassium hexacyanoferrate (III) using hydrogen peroxide as a reducing agent; this process was carried out according to the method of interfacial synthesis proposed by our scientific group more than 10 years ago [30].

### 2.3. Prussian Blue Nanoparticles Preparation

Prussian blue nanoparticles were synthesized in a 1:1 mixture of FeCl_3_ and K_3_[Fe(CN)_6_] (75 mM) dissolved in 0.1 M KCl with 0.1 M HCl under continuous ultrasonication. The precipitation process was initiated by adding 50 mM H_2_O_2_ as described in [16]. Dynamic light scattering performed with a Malvern Zetasizer Nano ZS (Malvern Instruments Ltd., Malvern, UK) was used for estimation of nanoparticle size distribution. UV/vis measurements in transmission mode were carried out using a Lambda 950 Spectrophotometer (PerkinElmer, Waltham, MA, USA). Prussian blue concentration in colloidal nanoparticle solutions was determined spectrophotometrically (ε_700nm_ (per PB unit cell) = 4.85 × 10^4^ M^−1^∙cm^−1^). An Eppendorf MiniSpin centrifuge (Hamburg, Germany) was used for nanoparticle separation. Ultrasonication of PBNP suspensions was carried out in a PSB-Gals (Moscow, Russia) ultrasonic bath. The obtained Prussian blue nanoparticle suspensions were stored at a pH of 1.1 and ultrasonicated prior to use. Changes in nanoparticle size and physicochemical properties were not observed during six months of storage at room temperature. For carbon/graphite ink modification, the resulting mixture was centrifuged at 13,000 rpm for 1 min, and dark blue precipitate was redispersed into di(propylenglycol)methyl ether (97%). The resulting suspension was used as addition for the carbon/graphite ink modification afterwards.

### 2.4. Biosensor Preparation

For biosensor preparation, screen-printed electrodes (both bulk- and surface-modified with PB) were used as a basis. Water–organic mixtures with high isopropanol content were used for enzyme immobilization. Perfluorosulfonated ionomer (PFSI) (Nafion analogue) and (γ-aminopropyl)triethoxysilane (APTES) were used as membrane-forming agents for glucose and lactate oxidase immobilization on working electrode surfaces, correspondingly. These membrane solutions were deposited through drop casting, followed by being air-dried for 24 h at 4 °C. GOx was immobilized from a water–isopropanol mixture (with solvent ratio 15:85) containing 0.3_vol_% PFSI as described in [31]. LOx was immobilized from a water–isopropanol mixture (with a solvent ratio of 1:9) with 1.5_vol_% of APTES according to [32]. The enzyme-containing membrane was formed on the electrode surface after solvent evaporation and the polycondensation process (in the case of the APTES-based membrane).

### 2.5. Electrochemical Measurements

Electrochemical investigations were carried out using PalmSens 4 potentiostat (PalmSens BV, The Netherlands). All the applied potentials mentioned in the paper refer to the internal Ag pseudo-reference electrode of the SPEs (potential of 0.25 V versus an SHE).

The response towards the analyte of the (bio)sensors was evaluated in batch mode by chronoamperometry. All the measurements were performed in 0.05 M phosphate buffer with 0.1 M KCl at pH 6.0 (5 mL) and at an applied potential of 0.0 mV. The calibration curves of hydrogen peroxide, glucose, and lactate were separately obtained with three different electrodes (using each electrode for all the concentration values tested) for each PBNP concentration in ink.

### 2.6. Control Serum Analysis

Standardized human serum samples were prepared by reconstituting lyophilized human serum with 5 mL of distilled water as described in the product instructions. Human serum was diluted 50 times by phosphate buffer solution prior to analysis. Analyte amperometric detection was carried out in the flow injection mode using the calibration curve obtained with standard solutions in a range of 0.01–0.1 mM and 0.02–0.4 mM for lactate and glucose, respectively.

## 3. Results

### 3.1. Bulk-Modified Hydrogen Peroxide Sensor

**Figure 1:**
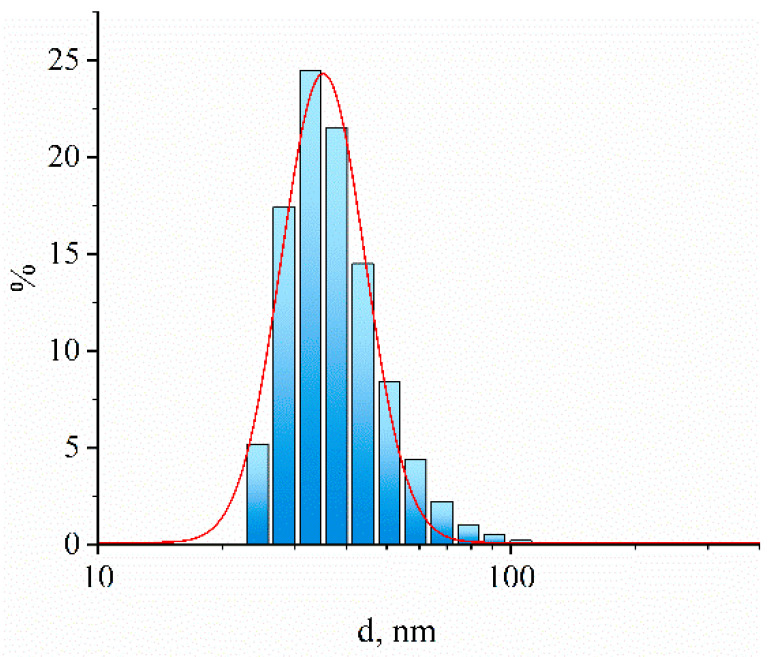
The example of hydrodynamic size distribution obtained by dynamic light scattering and fit to the log-normal distribution for Prussian Blue nanoparticles with the average diameter of 35 nm.

Prussian blue (PB) is known as the most advantageous electrocatalyst for hydrogen peroxide reduction; PB-modified electrodes are three orders of magnitude more active in H_2_O_2_ reduction and oxidation in neutral media and three orders of magnitude more selective for hydrogen peroxide reduction in the presence of oxygen compared to the most widely used platinum [9]. The advanced material can also be produced in the form of nanoparticles. In this work, PB nanoparticles with an average size of 35 nm (Figure 1) were used for working electrode bulk modification.

**Figure 2:**
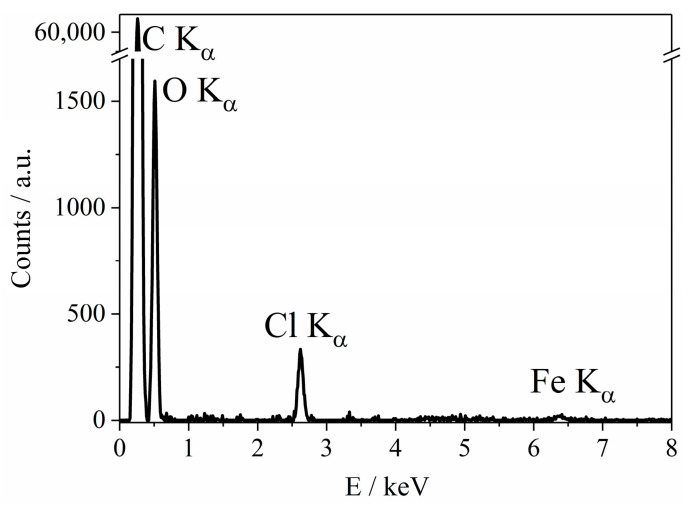
Energy dispersive X-ray (EDX) spectra of a PB nanoparticle-modified carbon screen-printed electrode (Prussian blue content 2.15 mg/g).

Carbon inks were modified by adding suspensions of nanoparticles with different concentrations to commercial carbon/graphite ink prior to printing the working electrode. Cyclic voltammograms of the resulting bulk-modified electrodes (Figure S2) show reversible peaks of Prussian blue reduction to Prussian white. The height of these peaks increases with an increase in the amount of PBNPs in the carbon/graphite ink. The amount of electroactive catalyst is rather low compared to surface-modified electrodes: 0.030 ± 0.008 nmol·cm^−2^ for the most concentrated suspensions of PBNPs compared to 1.5–2 nmol·cm^−2^ for surface-modified electrodes [33]. Energy-dispersive X-ray spectroscopy (EDX) measurements on bulk-modified electrodes (Figure 2) only indicate the presence of Prussian blue in the electrodes printed with the most concentrated samples. 

**Figure 3:**
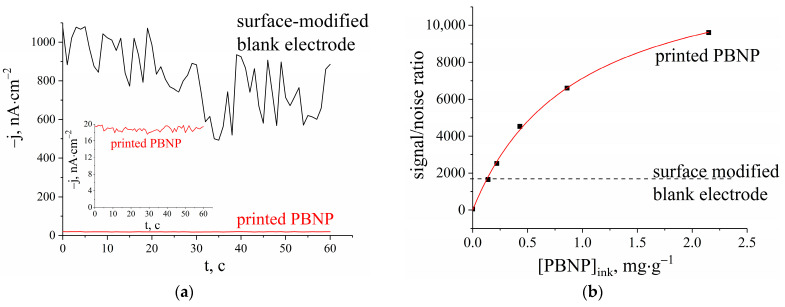
(**a**) The background and noise level for surface-modified blank electrodes (black line) and electrodes with printed Prussian blue nanoparticles (PBNP) (red line and inset); (**b**) signal-to-noise ratio dependance on Prussian blue nanoparticle concentration in carbon/graphite paste.

Lower amounts of the electrocatalyst obviously result in lower responses compared to those of surface-modified electrodes. However, as depicted in Figure 3a, bulk-modified electrodes show significantly reduced noise compared to surface-modified ones. The signal-to-noise ratio as a function of nanoparticle content in the carbon ink is displayed in Figure 3b and exhibits hyperbolic dependency tending toward the value of 13,800. Unfortunately, higher concentrations of PBNPs in carbon inks cannot be achieved due to printing restrictions. At the maximum achievable PBNP content, the signal-to-noise ratio surpasses 9500, which is approximately six times higher than that of surface-modified electrodes (Figure 3b).

**Figure 4:**
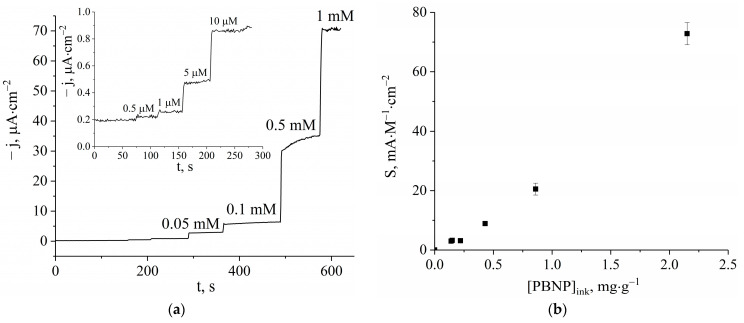
(**a**) The calibration curve for the printed hydrogen peroxide sensor with 2.15 mg/g of PBNPs in carbon/graphite ink; (**b**) sensitivity dependance on Prussian blue nanoparticle concentration in carbon/graphite ink.

The analytical performance characteristics of bulk-modified H_2_O_2_ sensors were studied in batch mode (Figure 4a). The sensitivity, detection limit, and linear range were studied for printed sensors with different printed Prussian blue nanoparticle concentrations. An almost linear relationship was observed between the sensitivity of the sensors and the amount of Prussian blue nanoparticles in the carbon/graphite paste used for printing (Figure 4b). The sensitivity of the sensor printed with the paste containing the maximum amount of PBNPs (2.15 mg/g) was 73 ± 4 mA M^−1^cm^−2^. The calibration graphs are linear in the range between 0.5 and 1000 µM, with a detection limit of 0.22 µM and a limit of quantification (LOQ) of 0.7 µM (Figure S3). Electrochemical treatment by cyclic voltammetry followed by annealing led to a twofold increase in sensor sensitivity. However, since this additional time-consuming procedure also resulted in similarly increased noise, it was excluded from further preparations.

In summary, despite their significantly lower sensitivity, the bulk-modified electrodes are characterized by a dramatically reduced (on average six times lower) signal-to-noise ratio, which provides a four times lower detection limit and a wider linear range (5 × 10^−7^–1 × 10^−3^ M) compared to the surface-modified Prussian blue sensors.

Thus, the novel approach allows for simplification of the manufacturing process, considerably decreasing the time and labor expenses required for sensor production. At the same time, the analytical performance characteristics of bulk-modified hydrogen peroxide sensors, in terms of both the limit of detection and linear range, are advantageous compared to those of surface-modified sensors.

### 3.2. Biosensors Based on Bulk-Modified Sensors

**Figure 5:**
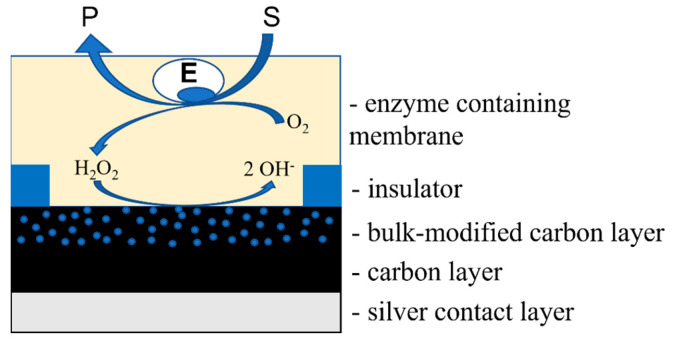
The construction scheme and functioning of the biosensor system made through the single printing step of carbon ink containing PBNPs.

A biosensor is made up of a biological recognition element that is directly linked to a transducer, which converts a chemical signal into a physical one. The developed bulk-modified sensors were used as transducers for oxidase-based biosensors. In these devices, the hydrogen peroxide produced during the enzyme-catalyzed reaction is detected through its reduction by the transducer (Figure 5). To create a biosensor, the appropriate oxidase enzyme is attached to the transducer surface. Our method involves exposing the enzyme to a water–organic mixture with a high concentration of organic solvent [31,32].

**Figure 6:**
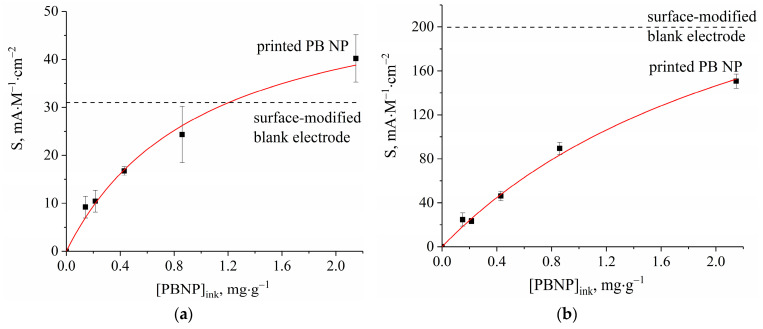
Sensitivity dependance on Prussian blue nanoparticle concentration in carbon/graphite ink for (**a**) glucose and (**b**) lactate biosensors based on printed hydrogen peroxide sensors.

Figure 6 shows the relationship between the sensitivity of the glucose (a) and lactate (b) biosensor and the PBNP amount in carbon/graphite ink. Biosensor sensitivity is increased with the increased amount of PBNPs. The data are approximated with hyperbolic functions. For both biosensors, sensitivity is at a similar level to that of surface-modified transducers (horizontal dash lines). In the case of the glucose biosensor, sensitivity with the highest amount of PBNP content in carbon/graphite ink was even higher than it was for biosensors based on surface-modified sensors (Figure 6a). Since noise levels are still reduced in the case of biosensors based on bulk-modified transducers, their limits of detection are on average four times lower for glucose- and two times lower for lactate-sensitive electrodes compared to similar biosensors based on surface-modified electrodes (Table 1). LOQ values were 5 µM and 1.6 µM for glucose and lactate biosensors based on bulk-modified electrodes, respectively, which were also lower than the LOQ values for biosensors based on surface-modified ones (25 and 4 µM).

The ratio of sensor-to-biosensor sensitivity in the case of surface-modified electrodes is known to be 15 and 2 for glucose and lactate biosensors, respectively. This ratio for biosensors based on bulk-modified sensors is found to be 1.8 and 0.5 for glucose and lactate biosensors, respectively (Table 1). However, the sensitivity of first-generation biosensors cannot exceed the sensitivity of sensors used as transducers.

To explain the higher sensitivity of the biosensor over that of the used transducer, we decided to find out whether deposition of the enzyme-containing membrane can affect the sensitivity of the H_2_O_2_ sensor. Hydrogen peroxide sensors made using the highest amount of PBNP content (2.15 mg/g) were treated with an isopropanol–water mixture (9:1), as well as with water–organic mixtures used for enzyme immobilization in the absence of the enzyme itself. The analytical performance of the resulting sensors was investigated. Treatment of the bulk-modified sensor with a water–organic mixture followed by subsequent drying for a day caused sensitivity to more than double (Table 2). After treatment with a membrane-forming mixture, the sensitivity increase was even higher: 3.5 times for PFSI and 4 times for APTES. Thus, taking into account transducer activation after deposition of the polymer membrane used for enzyme immobilization, the correct ratio of sensor-to-biosensor sensitivity is 6 and 2 for glucose and lactate biosensors, respectively. In conclusion, the efficiency of immobilized enzymes in biosensors is higher for bulk-modified ones, resulting in decreased sensor-to-biosensor sensitivity ratios.

One of the main advantages of enzyme-based biosensors is their selectivity. The developed biosensors show no response to 0.1 mM D-gluconic acid sodium salt, sodium tartrate, D-fructose, or D-mannose (Figure S4a,b) due to the high substrate specificity of enzymes used.

The elaborated biosensors were validated through lactate and glucose detection in human serum. The latter contained metabolites in normal and pathologic ranges (Figure S5). The detected glucose concentrations were 6.5 ± 0.1 and 12.8 ± 0.2 mM for normal and pathological serum samples, respectively. The obtained values are in agreement with the passport data: 5.8 ± 0.9 and 13 ± 2 mM. For lactate, the measured concentrations were 1.8 ± 0.1 mM in normal serum and 3.3 ± 0.2 mM in pathological serum, and the passport data were 1.6 ± 0.3 mM and 3.2 ± 0.6 mM, respectively. Current response linearly depended on glucose and lactate concentration in the range of 5.78 to 13.1 mM and 1.57 to 3.23 mM, correspondingly (Figure S6). The Pearson correlation coefficients were 0.994 and 0.971 for glucose and lactate. Thus, the developed biosensors can be used for serum analysis in clinical laboratories.

## 4. Conclusions

The attractive performance characteristics of catalytically synthesized PB nanoparticles [16] allow them to be denoted as artificial enzyme peroxidases—so called “nanozymes”. These nanozymes simplify the modification of electrode supports with Prussian blue, resulting in advanced sensors and biosensors [21,34].

However, the manufacturing process of PB-based sensors (Figure S2) is time-consuming and includes many steps, from electrode screen printing to deposition of the electrocatalyst with subsequent control of its amount on the electrode surface. Some stages are carried out for each electrode separately, thus complicating mass production. In this work, we suggest entrapment of PB nanoparticles into carbon ink, which would provide modification of the working electrode during screen printing. Accordingly, the manufacturing time for one sensor can be reduced from more than an hour and a half to several minutes. The use of Prussian blue nanoparticles (PBNPs) as a modifier provides advanced performance characteristics in the resulting H_2_O_2_ sensors. Thus, the limits of detection and quantification for hydrogen peroxide sensors manufactured by the new method are up to four times lower than those of surface-modified bulk electrodes due to the improved signal-to-noise ratio. This pattern is also observed for biosensors based on bulk-modified hydrogen peroxide sensors. Accordingly, the improvement in sensitivity is not the only way to provide the detection of low analyte concentrations. Another way may be the lowering of noise levels. 

In conclusion, using single printing step bulk-modified electrodes provides a simple, rapid, and easily mass-produced tool for oxidase-based biosensor development. Their attractive performance will ensure their wide use in (bio)sensorics and clinical laboratories.

## Figures and Tables

**Table 1:** Analytical performance of (bio)sensors based on printed electrodes.

PBNP Concentration, mg/g	H_2_O_2_ Sensor		Glucose Biosensor		Lactate Biosensor	
	S, mA·M^−1^·cm^−2^	LOD, M	S, mA·M^−1^·cm^−2^	LOD, M	S, mA·M^−1^·cm^−2^	LOD, M
0	0.030 ± 0.005	2.8 × 10^−5^	–	–	–	–
0.14	3.0 ± 0.5	9.1 × 10^−7^	9 ± 2	2.8 × 10^−6^	25 ± 6	8.0 × 10^−7^
0.22	3.1 ± 0.6	6.0 × 10^−7^	10 ± 2	1.6 × 10^−6^	23 ± 2	8.8 × 10^−7^
0.43	8.9 ± 0.4	3.3 × 10^−7^	16.8 ± 0.9	1.5 × 10^−6^	46 ± 4	6.0 × 10^−7^
0.86	21 ± 2	2.3 × 10^−7^	24 ± 6	1.3 × 10^−6^	89 ± 6	8.5 × 10^−7^
2.15	73 ± 4	2.2 × 10^−7^	40 ± 5	1.7 × 10^−6^	150 ± 10	5.5 × 10^−7^
surface-modified electrode	600 ± 20	8.9 × 10^−7^	31 ± 1	8.3 × 10^−6^	200 ± 30	1.3 × 10^−6^

**Table 2:** Analytical performance for printed hydrogen peroxide sensors with different pretreatments.

Pretreatment	Sensitivity, mA·M^−1^·cm^−2^
No pretreatment	73 ± 4
Isopropanol–water mixture (9:1)	160 ± 20
PFSI solution in isopropanol–water mixture	254 ± 6
APTES solution in isopropanol–water mixture	296 ± 2

## Data Availability

Not applicable.

## Supplementary Materials

The following supporting information can be downloaded at: https://www.mdpi.com/article/10.3390/bios13020250/s1, Figure S1: Manufacturing process for surface-modified blank electrodes. The dashed line highlights the part of the process required for printed PBNP electrodes; Figure S2: Cyclic voltammograms for printed hydrogen peroxide sensors with different concentrations of Prussian blue nanoparticles in carbon/graphite paste and control without any PBNPs in the carbon paste used; Figure S3: Calibration graph for the sensor based on bulk-modified electrodes (Prussian blue content of 2.15 mg/g) towards hydrogen peroxide in the batch mode (E = 0.00 V, phosphate buffer, pH 6.0.); Figure S4: Lactate (a) and glucose (b) biosensor responses towards an analyte and some saccharides and oxyacids in batch mode (all addition concentrations were 0.1 mM, E = 0.00 V, phosphate buffer, pH 6.0.); Figure S5: Glucose and lactate detection by biosensors based on printed hydrogen peroxide sensors with 0.43 mg/g of PBNPs in carbon/graphite paste; Figure S6: Glucose and lactate detection in human serum samples by biosensors based on printed hydrogen peroxide sensors with 2.15 mg/g of PBNPs in carbon/graphite paste versus sample passport data.

## References

[1] Niethammer P., Grabher C., Look A.T., Mitchison T.J. (2009). A tissue-scale gradient of hydrogen peroxide mediates rapid wound detection in zebrafish. Nature.

[2] Chen W., Cai S., Ren Q.Q., Wen W., Zhao Y.D. (2012). Recent advances in electrochemical sensing for hydrogen peroxide: A review. Analyst.

[3] Hawe A., Wiggenhorn M., van de Weert M., Garbe J.H., Mahler H.-C., Jiskoot W. (2012). Forced Degradation of Therapeutic Proteins. J. Pharm. Sci..

[4] Seresirikachorn K., Phoophiboon V., Chobarporn T., Tiankanon K., Aeumjaturapat S., Chusakul S., Snidvongs K. (2021). Decontamination and reuse of surgical masks and N95 filtering facepiece respirators during the COVID-19 pandemic: A systematic review. Infect. Control Hosp. Epidemiol..

[5] Kumkrong P., Scoles L., Brunet Y., Baker S. (2021). Determination of hydrogen peroxide on N95 masks after sanitization using a colorimetric method. Methodsx.

[6] Ivanova A.S., Merkuleva A.D., Andreev S.V., Sakharov K.A. (2019). Method for determination of hydrogen peroxide in adulterated milk using high performance liquid chromatography. Food Chem..

[7] Liu S.G., Liu S., Yang S., Zhao Q., Deng J., Shi X. (2022). A facile fluorescent sensing strategy for determination of hydrogen peroxide in foods using a nanohybrid of nanoceria and carbon dots based on the target-promoted electron transfer. Sens. Actuators B Chem..

[8] Xing L., Zhang W., Fu L., Lorenzo J.M., Hao Y. (2022). Fabrication and application of electrochemical sensor for analyzing hydrogen peroxide in food system and biological samples. Food Chem..

[9] Karyakin A.A. (2017). Advances of Prussian blue and its analogues in (bio)sensors. Curr. Opin. Electrochem..

[10] Piermarini S., Migliorelli D., Volpe G., Massoud R., Pierantozzi A., Cortese C., Palleschi G. (2013). Uricase biosensor based on a screen-printed electrode modified with Prussian blue for detection of uric acid in human blood serum. Sens. Actuators B Chem..

[11] Gurban A.-M., Noguer T., Bala C., Rotariu L. (2008). Improvement of NADH detection using Prussian blue modified screen-printed electrodes and different strategies of immobilisation. Sens. Actuators B Chem..

[12] Harrad L.E., Amine A. (2016). Amperometric biosensor based on prussian blue and nafion modified screen-printed electrode for screening of potential xanthine oxidase inhibitors from medicinal plants. Enzyme Microb. Technol..

[13] Yashina E.I., Borisova A.V., Karyakina E.E., Shchegolikhina O.I., Vagin M.Y., Sakharov D.A., Tonevitsky A.G., Karyakin A.A. (2010). Sol−Gel Immobilization of Lactate Oxidase from Organic Solvent: Toward the Advanced Lactate Biosensor. Anal. Chem..

[14] Khumngern S., Jirakunakorn R., Thavarungkul P., Kanatharana P., Numnuam A. (2021). A highly sensitive flow injection amperometric glucose biosensor using a gold nanoparticles/polytyramine/Prussian blue modified screen-printed carbon electrode. Bioelectrochemistry.

[15] Ulasova E.A., Micheli L., Vasii L., Moscone D., Palleschi G., Vdovichev S.V., Zorin A.V., Krutovertsev S.A., Karyakina E.E., Karyakin A.A. (2003). Flow-Injection Analysis of Residual Glucose in Wines Using a Semiautomatic Analyzer Equipped with a Prussian Blue-Based Biosensor. Electroanalysis.

[16] Komkova M.A., Karyakina E.E., Karyakin A.A. (2018). Catalytically Synthesized Prussian Blue Nanoparticles Defeating Natural Enzyme Peroxidase. J. Am. Chem. Soc..

[17] Komkova M.A., Karyakin A.A. (2022). Prussian blue: From advanced electrocatalyst to nanozymes defeating natural enzyme. Microchim. Acta.

[18] Komkova M.A., Ibragimova O.A., Karyakina E.E., Karyakin A.A. (2021). Catalytic Pathway of Nanozyme “Artificial Peroxidase” with 100-Fold Greater Bimolecular Rate Constants Compared to Those of the Enzyme. J. Phys. Chem. Lett..

[19] Komkova M.A., Zarochintsev A.A., Karyakin A.A. (2022). Nanozymes ‘artificial peroxidase’ in reduction and detection of organic peroxides. J. Electroanal. Chem..

[20] Banerjee S., Sarkar P., Turner A.P. (2013). Amperometric biosensor based on Prussian Blue nanoparticle-modified screen-printed electrode for estimation of glucose-6-phosphate. Anal. Biochem..

[21] Vokhmyanina D.V., Andreeva K.D., Komkova M.A., Karyakina E.E., Karyakin A.A. (2020). ‘Artificial peroxidase’ nanozyme—Enzyme based lactate biosensor. Talanta.

[22] Aller-Pellitero M., Fremeau J., Villa R., Guirado G., Lakard B., Hihn J.-Y., del Campo F.J. (2019). Electrochromic biosensors based on screen-printed Prussian Blue electrodes. Sens. Actuators B Chem..

[23] Cinti S., Arduini F., Moscone D., Palleschi G., Killard A.J. (2014). Development of a Hydrogen Peroxide Sensor Based on Screen-Printed Electrodes Modified with Inkjet-Printed Prussian Blue Nanoparticles. Sensors.

[24] Cinti S., Arduini F., Vellucci G., Cacciotti I., Nanni F., Moscone D. (2014). Carbon black assisted tailoring of Prussian Blue nanoparticles to tune sensitivity and detection limit towards H_2_O_2_ by using screen-printed electrode. Electrochem. Commun..

[25] Cinti S., Arduini F., Moscone D., Palleschi G., Gonzalez-Macia L., Killard A.J. (2015). Cholesterol biosensor based on inkjet-printed Prussian blue nanoparticle-modified screen-printed electrodes. Sens. Actuators B Chem..

[26] da Cruz F.S., de Souza Paula F., Franco D.L., dos Santos W.T.P., Ferreira L.F. (2017). Electrochemical detection of uric acid using graphite screen-printed electrodes modified with Prussian blue/poly(4-aminosalicylic acid)/Uricase. J. Electroanal. Chem..

[27] Hirst N., Hazelwood L., Jayne D., Millner P. (2013). An amperometric lactate biosensor using H_2_O_2_ reduction via a Prussian Blue impregnated poly(ethyleneimine) surface on screen printed carbon electrodes to detect anastomotic leak and sepsis. Sens. Actuators B Chem..

[28] Sekar N.C., Shaegh S.A.M., Gary N.S.H., Liya G., Ngin T.S. (2014). A paper-based amperometric glucose biosensor developed with Prussian Blue-modified screen-printed electrodes. Sens. Actuators B Chem..

[29] O’Halloran M.P., Pravda M., Guilbault G.G. (2001). Prussian Blue bulk modified screen-printed electrodes for H_2_O_2_ detection and for biosensors. Talanta.

[30] Borisova A.V., Karyakina E.E., Cosnier S., Karyakin A. (2009). Current-Free Deposition of Prussian Blue with Organic Polymers: Towards Improved Stability and Mass Production of the Advanced Hydrogen Peroxide Transducer. Electroanalysis.

[31] Karyakin A.A., Kotel’Nikova E.A., Lukachova L.V., Karyakina E.E., Wang J. (2002). Optimal Environment for Glucose Oxidase in Perfluorosulfonated Ionomer Membranes: Improvement of First-Generation Biosensors. Anal. Chem..

[32] Pribil M.M., Cortés-Salazar F., Andreyev E.A., Lesch A., Karyakina E.E., Voronin O.G., Girault H.H., Karyakin A.A. (2014). Rapid optimization of a lactate biosensor design using soft probes scanning electrochemical microscopy. J. Electroanal. Chem..

[33] Komkova M.A., Karpova E.V., Sukhorukov G.A., Sadovnikov A.A., Karyakin A.A. (2016). Estimation of continuity of electroactive inorganic films based on apparent anti-Ohmic trend in their charge transfer resistance. Electrochim. Acta.

[34] Komkova M.A., Zarochintsev A.A., Karyakina E.E., Karyakin A.A. (2020). Electrochemical and sensing properties of Prussian Blue based nanozymes “artificial peroxidase”. J. Electroanal. Chem..

