# Supplementary material for: Single Printing Step Prussian Blue Bulk-Modified Transducers for Oxidase-Based Biosensors
Source: Biosensors (Basel). 2023 Feb 9;13(2):250. doi: 10.3390/bios13020250 (PMC9953944; doi:10.3390/bios13020250)
Supplement: Supplementary file 1 [file biosensors-13-00250-s001.zip › biosensors-2067349-supplementary.pdf]

biosensors

Supplementary Material

Single Printing Step Prussian Blue Bulk-Modified Transducers for Oxidase-Based Biosensors

Darya Vokhmyanina *, Elena Daboss, Olesya Sharapova, Mariia Mogilnikova and Arkady Karyakin

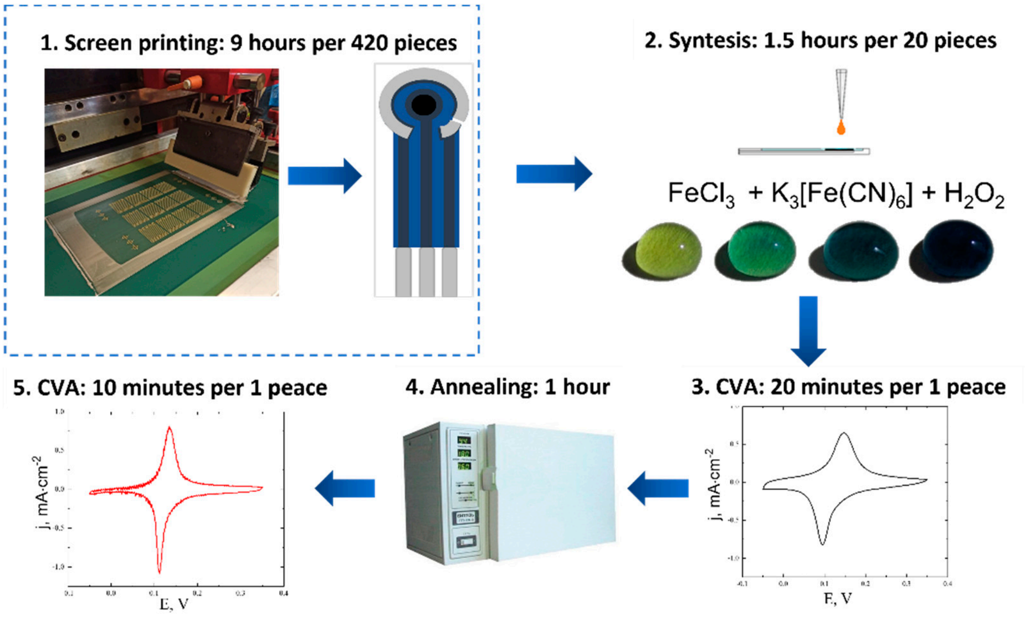

Figure S1. Manufacturing process for surface modified blank electrodes. The dashed line highlights part of the process required for printed Prussian blue nanoparticles (PBNP) electrodes.

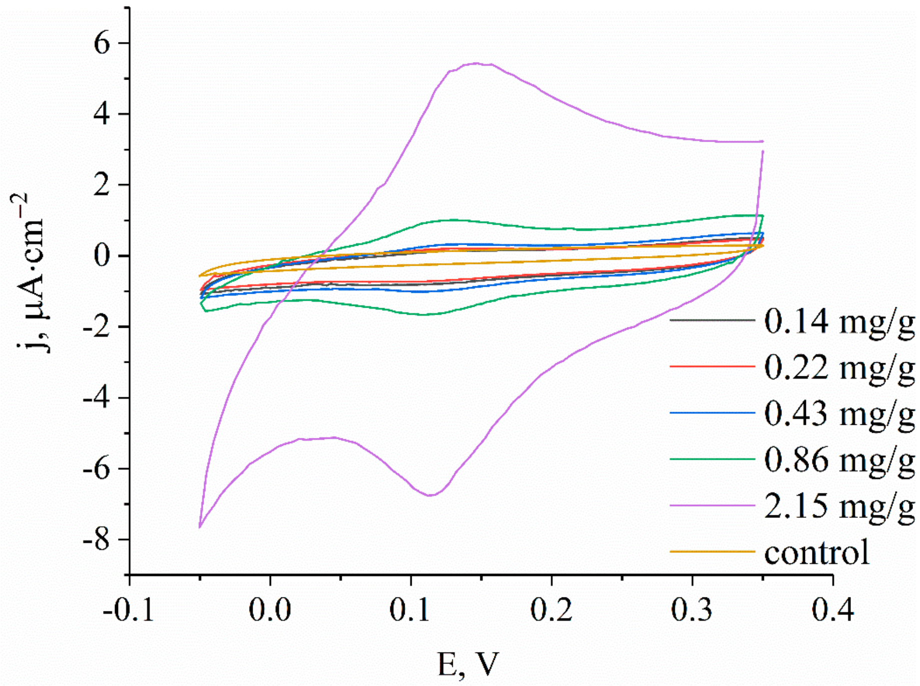

Figure S2. Cyclic voltammograms for printed hydrogen peroxide sensors with different concentrations of Prussian Blue nanoparticles in carbon/graphite paste and control without any PBNPs in carbon paste used.

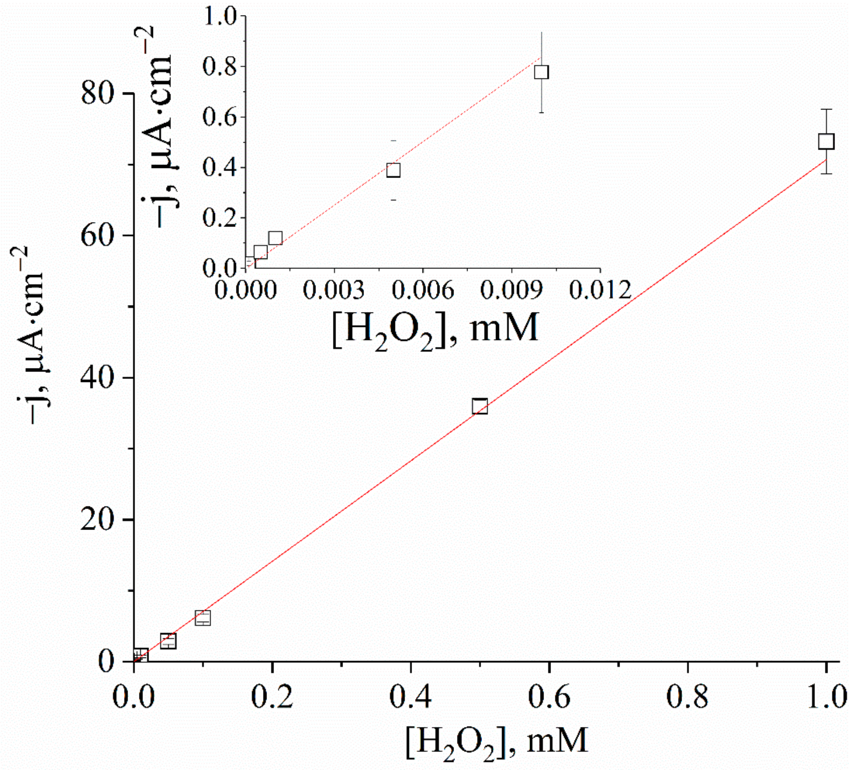

Figure S3. Calibration graph for the sensor based on bulk-modified electrodes (Prussian Blue content 2.15 mg/g) towards hydrogen peroxide in batch mode (E = 0.00 V, phosphate buffer, pH 6.0.).

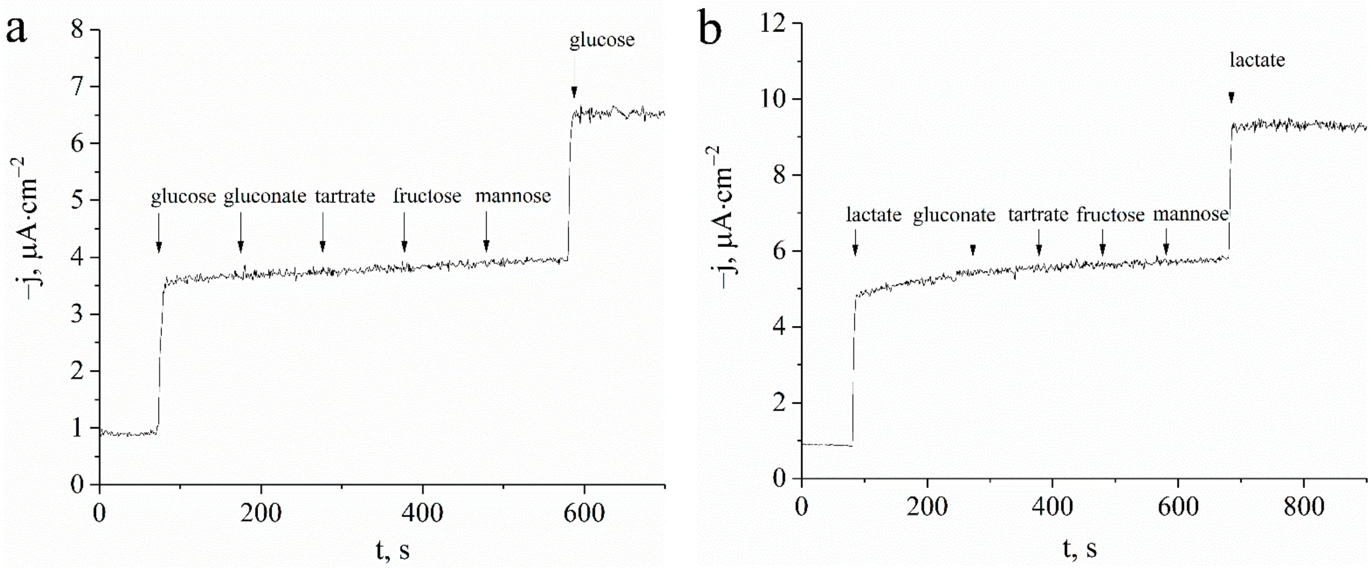

Figure S4. Lactate (a) and glucose (b) biosensors responses towards analyte and some saccharides and oxyacids in batch mode (all additions concentrations 0.1 mM, E = 0.00 V, phosphate buffer, pH 6.0.).

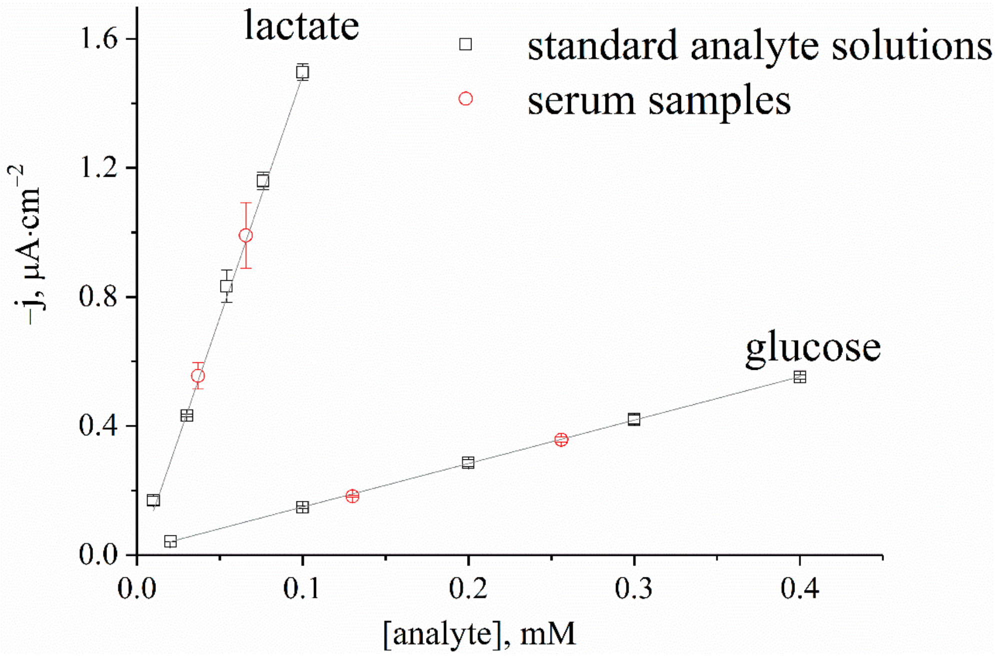

Figure S5. The glucose and lactate detection by biosensors based on printed hydrogen peroxide sensors with 0.43 mg/g PBNP in carbon/graphite paste.

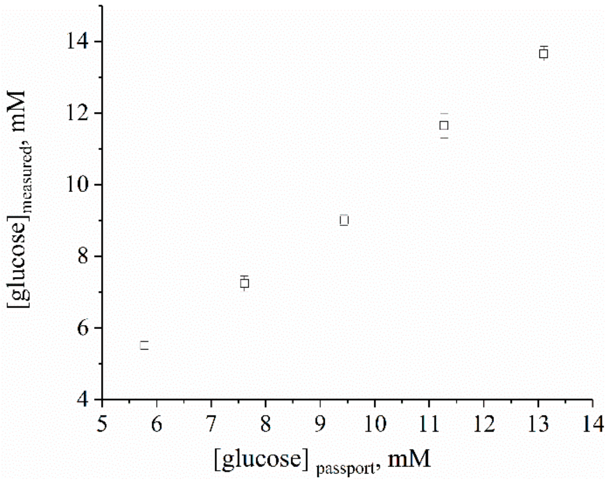

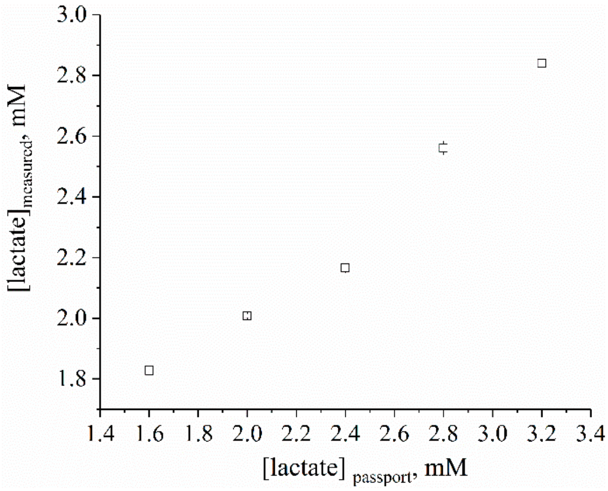

Figure S6. The glucose and lactate detection in human serum samples by biosensors based on printed hydrogen peroxide sensors with 2.15 mg/g PBNP in carbon/graphite paste versus sample passport data.

Data from sample passport were used as "x" for standard human serum samples. The Pearson correlation coefficients were 0.994 and 0.971 for glucose and lactate biosensors, correspondingly.
